# Comparison of adiposity anthropometric indices and their associations with visceral fat levels determined by bioelectrical impedance analysis among diabetic patients

**DOI:** 10.1038/s41598-022-22848-z

**Published:** 2022-10-24

**Authors:** Lawrence Sena Tuglo

**Affiliations:** Department of Nutrition and Dietetics, School of Allied Health Sciences, University of Health and Allied Sciences, Ho, Ghana

**Keywords:** Physiology, Diseases, Health care, Medical research, Risk factors

## Abstract

Visceral fat (VF) and its effect on metabolic disorders have been extensively studied; nevertheless, there is a need for a simple and reliable index to equally assess VF in low-resource settings. This multihospital-based study was designed to compare the five adiposity anthropometric indices and their associations with VF levels determined by bioelectrical impedance analysis as the reference standard among diabetic patients. A pretested questionnaire was used to collect anthropometric, biochemical and hemodynamic data from 473 diabetic patients. Regression analysis was performed to determine the associations between the five adiposity anthropometric indices and VF levels. Receiver operating characteristic (ROC) curves were used to confirm the predictive capacities of the five adiposity anthropometric indices with VF levels. The waist-to-height ratio WHtR showed the greatest ROC value [area under the curve (AUC) = 0.745, *p* ˂0.001] in identifying diabetic patients with high VF levels compared to body mass index BMI [AUC = 0.584, *p* = 0.047], waist circumference WC [AUC = 0.723, *p* ˂0.001], hip circumference HC [AUC = 0.647, *p* ˂0.001] and waist-to-hip ratio WHR [AUC = 0.711, *p* ˂0.001]. Likewise, the regression analysis of WHtR and VF levels revealed the strongest association [unadjusted odds ratio (UOR) = 21.49, *p* < 0.001] compared to BMI [UOR = 6.77, *p* = 0.008], WC [UOR = 6.37, *p* < 0.001], HC [UOR = 5.93, *p* = 0.002] and WHR [UOR = 13.17, *p* < 0.001]. The optimal cut-off values to identify diabetic patients with high VF levels were > 0.5 for WHtR, > 25.7 kg/m2 for BMI, > 80.5 cm for WC, > 95.5 cm for HC and > 0.82 for WHR. WHtR was shown to have overpowered BMI, HC, WC and WHR in identifying diabetic patients with high VF levels. Therefore, the Ghana Health Service could recommend WHtR as a better diagnostic index for assessing VF levels due to its high predictive capacity.

## Introduction

Diabetic patients usually possess greater quantities of visceral, subcutaneous, total, and intermuscular adipose tissues than healthy people^1–4^. Studies have found an association between high VF and cardiovascular diseases, insulin resistance, dyslipidemia and glucose intolerance in diabetic patients^2,3,5^. This predisposes diabetic patients to several diabetes complications, metabolic abnormalities, and cardiometabolic diseases^2–4,6^. Based on these findings, it has become necessary to assess an individual’s body composition, particularly percentages of fat and muscle.

The use of computed tomography (CT) and magnetic resonance imaging (MRI) in measuring VF provides valid and reliable estimates^2,7,8^. However, they are expensive and require professional skills and exposure to radiation^7,8^. These are not favourable for global and daily use. There is another device known as bioelectrical impedance analysis (BIA) for estimating body composition (e.g., body fat and muscle mass)^9^. Compared to CT and MRI, it is safest, less expensive, noninvasive and simple^2,7^. Nevertheless, it also requires little competency to operate. In addition, BIA outputs are affected by several factors, such as ethnicity, environment, phase of the menstrual cycle, dehydration, and underlying medical conditions^9,10^.

Meanwhile, there should be a simple method that is less expensive and reliable to equally estimate VF levels in low-resource settings for clinical practice and epidemiological studies. Several studies^2–4,10–14^, including the World Health Organization (WHO)^15,16^, have recommended the use of BMI, HC and WC for assessing adiposity. Another indicator, such as WHR, has also been proposed due to the effect of age, gender, and ethnic disparities on BMI^2–4,14^ and the low accuracy in identifying central obesity^2–4,10^ in some populations. Recently, a new index called WHtR has gained popularity and has been used extensively for assessing the risks of cardiometabolic diseases, adiposity and metabolic syndrome^10,17,18^. It provides accurate information about adiposity status by considering height, gender and ethnicity disparities^10,19^.

The majority of these studies that reported the cut-off values of the abovementioned anthropometric indices are largely based on data from Asian, European and American populations^3,10,20–22^. These might not be accurately useful to other populations. Therefore, it is necessary to find appropriate cut-off values of each anthropometric index for the assessment of adiposity in Africa with different ethnicities, especially in Sub-Saharan Africa (SSA). Henceforth, considering the burden of visceral adiposity in Ghana among diabetic patients^1,2^, this multihospital-based study was designed to compare the five adiposity anthropometric indices and their associations with VF levels determined by BIA as the reference standard to identify the best diagnostic index for assessing VF levels among diabetic patients in the Volta Region, Ghana.

## Materials and methods

### Study design and setting

This was a multihospital-based cross-sectional study conducted in the Volta Region, Ghana, between September 2019 and December 2019. Details of the study methodology, first^23^ and second papers^24^, have been published. The final paper is different and unique in terms of content. It focused on a “comparison of adiposity anthropometric indices and their associations with VF levels determined by BIA among diabetic patients”. The study protocol with an identification number (UHAS-REC No: A1 [16] 19–20) was approved by the Research Ethics Committee (REC) of the University of Health and Allied Sciences (UHAS), Ho, Volta Region, Ghana.

### Study population and subject selection

Diabetic patients who visited the designated hospitals during their appointments were recruited through a systematic sampling technique^23^. Patients who were medically stable and willing to participate in the study were interviewed face-to-face^23^. Sick patients who were unable to talk, those who disagreed to participate and gestational diabetic patients were excluded from the study^23^. A total of 473 diabetic patients were enrolled and were determined based on the study population (N = 1256) using Yamane's formula; n = N/1 + N(e^2^)^25^, where n = sample size, N = population size, e = margin of error (5%), plus 10% nonresponse.

### Anthropometric, biochemical, hemodynamic and BIA measurements

The physical assessments of diabetic patients included height, body weight, WC, HC, fasting blood sugar (FBS), systolic blood pressure (SBP) and diastolic blood pressure (DBP). Weight was measured in a patient lightly dressed without shoes to the nearest 0.1 kg using a portable digital scale. Height was measured to the nearest 0.1 cm according to a standard method using a tape measure attached firmly to the wall. WC was measured at the umbilical position during the exhalation state while standing with a light dress and recorded to the nearest 0.1 cm. The HC was measured around the widest circumference of the buttock and recorded to the nearest 0.1 cm. The FBS, SBP and DBP data were retrieved from the patients' folders. BMI was calculated as weight (kg) divided by squared height (m^2^). WHR and WHtR were calculated by dividing WC (cm) by HC (cm) and height (cm), respectively. The BIA (Omron BF-511; Omron Healthcare Co., Ltd., Kyoto, Japan) was used to calculate the impedance of the body of each diabetic patient by imputing the age, gender and height^9^. The BIA estimates the body composition by allowing a small painless low-level electrical current through different kinds of body tissue^9^. The procedures were as follows: each patient was asked to step barefoot onto the BIA while on the ground and hold the display unit, which was the BIA handlers with both hands. While standing vertically, the patient was asked to extend the arms parallel to the floor at the same level as the shoulder. The BIA generated the VF value for each diabetic patient, which was used as the reference standard in the study.

### Anthropometrics and VF classifications

The BMI values were categorized according to the WHO classification: < 18.5 kg/m^2^ (underweight), 18.5 to 24.9 kg/m^2^ (normal weight), 25 to 29.9 kg/m^2^ (overweight) and ≥ 30 kg/m^2^ (obese)^15^. The values of WC, HC, WHR, and WHtR were classified into four quartiles (1^st^ quartile = Q1, 2nd quartile = Q2, 3rd quartile = Q3 and 4th quartile = Q4) for regression analysis used by previous studies^3,4^. For WC, Q1 (< 76 cm), Q2 (76–84 cm), Q3 (85–94 cm) and Q4 (≥ 95 cm). For HC, Q1 (< 92 cm), Q2 (92–99 cm), Q3 (100–108 cm) and Q4 (≥ 109 cm). For WHR, Q1 (< 0.79), Q2 (0.79–0.85), Q3 (0.86–0.90) and Q4 (≥ 0.91). For WHtR, Q1 (< 0.47), Q2 (0.47–0.52), Q3 (0.53–0.57) and Q4 (≥ 0.58). The VF values generated by the BIA were classified according to Omron BF-511; Omron Healthcare Co., Ltd., Kyoto, Japan manual as follows: (1–9) was “Normal”, (10–14) was “High” and (15–30) was “Very High” regardless of gender^9^. However, in this study, VF values were reclassified into two categories: (1–9) was considered “Normal” and (10–30) was considered “High” and used as the reference standard.

### Data collection and quality assurance

The data collection and measurements were performed by trained research assistants after validating and pretesting the structured questionnaire^23^. Data were collected systematically by dividing the study population (1256) with the estimated sample size (334) to obtain the fraction (x), x = N/n = 1256/334 = 3.76. The administration of the questionnaire and measurements commenced from the 1st diabetic patient to the 5th until the last patient was served^23^.

### Data analysis

Analysis was performed by SPSS version 25.00. Categorical data were equated using Fisher's exact test or the Chi-squared test. Student’s t-test for the comparison of continuous data. Three models were adapted for the regression analysis used in previous studies^3,4,13^. Model 1^**†**^ was unadjusted. Model 2^**‡**^ was adjusted for SBP and DBP. Model 3^**ұ**^ was adjusted for gender, age, weight, height and FBS^3,4,13^. Regression analysis was performed to determine the associations between the five adiposity anthropometric indices and VF levels. The regression analysis focused only on the odds ratio (OR) for BMI (normal versus obese) and WC, HC, WHR and WHtR (1st quartile versus 4th quartile)^3,4^. ROC curves were used to confirm the predictive capacities of the five adiposity anthropometric indices with VF levels^7,26^. Youden’s index was calculated to determine the optimum cut-off points of the five adiposity anthropometric indices^26,27^. A *p* value < 0.05 was considered significant in all analyses.

### Ethics approval and consent to participate

Ethical clearance was obtained from the Research Ethics Committee (REC) of the University of Health and Allied Sciences (UHAS), Ho, with ethical approval number (UHAS-REC No: A.1[16]19–20). Additionally, approval was obtained from the health facilities administration. For this study, informed consent was obtained from all subjects and/or their legal guardian(s) before the aims of the study were explained to them in the languages they understood. All methods were carried out following relevant guidelines and regulations. Privacy and confidentiality were confirmed by filling out the questionnaire singly and without special credentials.

## Results

### Baseline characteristics of diabetic patients stratified by visceral fat levels

The prevalence of high VF levels was 88.8% (n = 420), determined by BIA as the reference standard among 473 diabetic patients enrolled in the study. There were more females than males, but the difference was not statistically significant (*p* = 0.485). The total average age was 50.8 ± 0.7 years. No significant differences were found between the VF levels for age, weight, height and FBS (*p* > 0.05). Other anthropometric and hemodynamic parameters were statistically significant (*p* < 0.05). The means for BMI, WC, HC, WHR, WHtR, SBP and DBP were significantly higher among diabetic patients with high VF levels than among those with normal VF levels. According to the classification of WC, HC, WHR and WHtR into quartiles, when compared to the 1^st^ quartile, greater proportions of diabetic patients with high VF levels were found in the 4^th^ quartile: WC (19.5 vs. 30.0%), HC (23.1 vs. 27.4%), WHR (19.5 vs. 32.1%) and WHtR (17.9 vs. 29.5%) (see Table 1).

**Table 1:** Baseline characteristics of diabetic patients stratified by visceral fat levels.

Parameter	Total	Normal	High	*p*-value
Gender (F)	349 (73.8)	37 (69.8)	312 (74.3)	0.485
Age (years)	50.80 ± 0.68	53.15 ± 1.82	50.50 ± 0.73	0.180
Weight (kg)	71.58 ± 0.66	69.91 ± 1.91	71.79 ± 0.71	0.358
Height (m)	1.63 ± 0.00	1.66 ± 0.01	1.63 ± 0.00	0.105
BMI (kg/m^2^)	26.82 ± 0.24	25.55 ± 0.64	26.98 ± 0.25	0.042
**BMI classification**				0.038
Underweight	22 (4.7)	5 (9.4)	17 (4.0)	
Normal	119 (25.2)	16 (30.2)	103 (24.5)	
Overweight	236 (49.9)	28 (52.8)	208 (49.5)	
Obese	96 (20.3)	4 (7.5)	92 (21.9)	
WC (cm)	86.12 ± 0.633	77.32 ± 1.47	87.23 ± 0.67	< 0.001
**WC according to quartile**				< 0.001
1st quartile	111 (23.5)	29 (54.7)	82 (19.5)	
2nd quartile	117 (24.7)	13 (24.5)	104 (24.8)	
3rd quartile	112 (23.7)	4 (7.5)	108 (25.7)	
4th quartile	133 (28.1)	7 (13.2)	126 (30.0)	
HC (cm)	101.02 ± 0.66	95.94 ± 1.43	101.66 ± 0.72	0.001
**HC according to quartile**				0.007
1st quartile	117 (24.7)	20 (37.7)	97 (23.1)	
2nd quartile	119 (25.2)	16 (30.2)	103 (24.5)	
3rd quartile	118 (24.9)	13 (24.5)	105 (25.0)	
4th quartile	119 (25.2)	4 (7.5)	115 (27.4)	
WHR	0.85 ± 0.00	0.81 ± 0.01	0.86 ± 0.00	< 0.001
**WHR according to quartile**				< 0.001
1st quartile	106 (22.4)	24 (45.3)	82 (19.5)	
2nd quartile	130 (27.5)	16 (30.2)	114 (27.1)	
3rd quartile	99 (20.9)	10 (18.9)	89 (21.2)	
4th quartile	138 (29.2)	3 (5.7)	135 (32.1)	
WHtR	0.53 ± 0.00	0.47 ± 0.01	0.54 ± 0.00	< 0.001
**WHtR according to quartile**				< 0.001
1st quartile	101 (21.4)	26 (49.1)	75 (17.9)	
2nd quartile	135 (28.5)	15 (28.3)	120 (28.6)	
3rd quartile	111 (23.5)	10 (18.9)	101 (24.0)	
4th quartile	126 (26.6)	2 (3.8)	124 (29.5)	
FBS (mmol/L)	9.95 ± 0.24	8.99 ± 0.65	10.07 ± 0.25	0.125
SBP (mmHg)	120.66 ± 0.93	114.15 ± 1.71	121.48 ± 1.02	< 0.001
DBP (mmHg)	76.69 ± 0.48	72.83 ± 1.08	77.18 ± 0.52	< 0.001

Data are presented as frequencies with percentages in parentheses and means ± standard errors of the means. Where appropriate, categorical data were compared using Fisher's exact test or the Chi-squared test, and continuous data were compared using Student’s t-test.

### Regression analysis showing the associations of BMI, WC, HC, WHR and WHtR with VF levels in diabetic patients

The study showed that BMI, WC, HC, WHR and WHtR were significantly associated with VF levels. In Model 1^**†**^, diabetic patients classified as obese were more likely to possess high VF levels compared to normal BMI [(UOR) = 6.77, (95% Cl 1.65–27.79), *p* = 0.008], those in the 4th quartile compared to the 1st quartile were more likely to possess high VF levels, WC [UOR = 6.37, (95% Cl 2.66–15.21), *p* < 0.001], HC [UOR = 5.93, (95% Cl 1.96–17.93), *p* = 0.002], WHR [UOR = 13.17, (95% Cl 3.85–45.11), *p* < 0.001], and WHtR [UOR = 21.49, (95% Cl 4.96–93.16), *p* < 0.001]. In Model 2^**‡**^, obese diabetic patients were more likely to possess high VF levels than those with normal BMI [adjusted odds ratio (AOR) = 5.16, (95%, Cl 1.22–21.87), *p* = 0.026], and diabetic patients in the 4th quartile were more likely to possess high VF levels than those in the 1st quartile, WC [AOR = 5.24, (95%, Cl 2.08–13.23), *p* < 0.001], HC [AOR = 4.85, (95% Cl 1.57–15.00), *p* = 0.006], WHR [AOR = 11.23, (95% Cl 3.23–38.99), *p* < 0.001] and WHtR [AOR = 18.68, (95% Cl 4.07–85.73), *p* < 0.001]. In Model 3^**ұ**^, this study showed that WHtR has the greatest predictive capacity in identifying diabetic patients with high VF levels [Q4 vs. Q1, (AOR) = 51.15, (95% Cl 9.22–283.82) *p* < 0.001] followed by BMI [AOR = 43.31, (95% Cl 1.91–981.73), *p* = 0.018], WC [AOR = 20.41, (95% Cl 5.66–73.64), *p* < 0.001], WHR [AOR = 12.45, (95% Cl 3.59–43.24), *p* < 0.001) and HC [AOR = 11.94, (95% Cl 2.84–50.22), *p* = 0.001] (see Table 2).

**Table 2:** Regression analysis showing the associations of BMI, WC, HC, WHR and WHtR with VF levels in diabetic patients.

Parameter and model	Adiposity anthropometric indices classifications				*p* value
BMI	Underweight	Normal	Overweight	Obese	
Model 1^†^	1.89 (0.61–5.85)	1	2.19 (0.75–6.39)	6.77 (1.65–27.79)	0.008
Model 2^‡^	1.81 (0.58–5.72)	1	1.92 (0.65–5.72)	5.16 (1.22–21.87)	0.026
Model 3^ұ^	3.01 (0.77–11.84)	1	6.11 (0.92–40.43)	43.31 (1.91–981.73)	0.018
WC	1st quartile	2nd quartile	3rd quartile	4th quartile	
Model 1^†^	1	2.83 (1.38–5.79)	9.55 (3.23–28.23)	6.37 (2.66–15.21)	< 0.001
Model 2^‡^	1	2.65 (1.29–5.47)	8.53 (2.84–25.57)	5.24 (2.08–13.23)	< 0.001
Model 3^ұ^	1	4.88 (2.15–11.06)	17.54 (5.38–57.20)	20.41 (5.66–73.64)	< 0.001
HC	1st quartile	2nd quartile	3rd quartile	4th quartile	
Model 1^†^	1	1.33 (0.65–2.71)	1.67 (0.79–3.53)	5.93 (1.96–17.93)	0.002
Model 2^‡^	1	1.32 (0.64–2.71)	1.39 (0.64–3.01)	4.85 (1.57–15.00)	0.006
Model 3^ұ^	1	1.51 (0.71–3.22)	2.59 (1.05–6.36)	11.94 (2.84–50.22)	0.001
WHR	1st quartile	2nd quartile	3rd quartile	4th quartile	
Model 1^†^	1	2.09 (1.04–4.17)	2,61 (1.18–5.78)	13.17 (3.85–45.11)	< 0.001
Model 2^‡^	1	1.98 (0.99–3.99)	2.16 (0.95–4.90)	11.23 (3.23–38.99)	< 0.001
Model 3^ұ^	1	2.23 (1.10–4.54)	2.84 (1.23–6.55)	12.45 (3.59–43.24)	< 0.001
WHtR	1st quartile	2nd quartile	3rd quartile	4th quartile	
Model 1^†^	1	2.77 (1.38–5.57)	3.50 (1.59–7.70)	21.49 (4.96–93.16)	< 0.001
Model 2^‡^	1	2.62 (1.27–5.40)	3.31 (1.47–7.45)	18.68 (4.07–85.73)	< 0.001
Model 3^ұ^	1	4.02 (1.86–8.70)	6.27 (2.40–16.37)	51.15 (9.22–283.82)	< 0.001

^†^Unadjusted. ^‡^Adjusted for SBP and DBP. ^ұ^Adjusted for gender, age, weight, height and FPS. 1: reference.

### ROC curve analysis for comparison of BMI, WC, HC, WHR and WHtR in determining VF levels in diabetic patients

**Figure 1:**
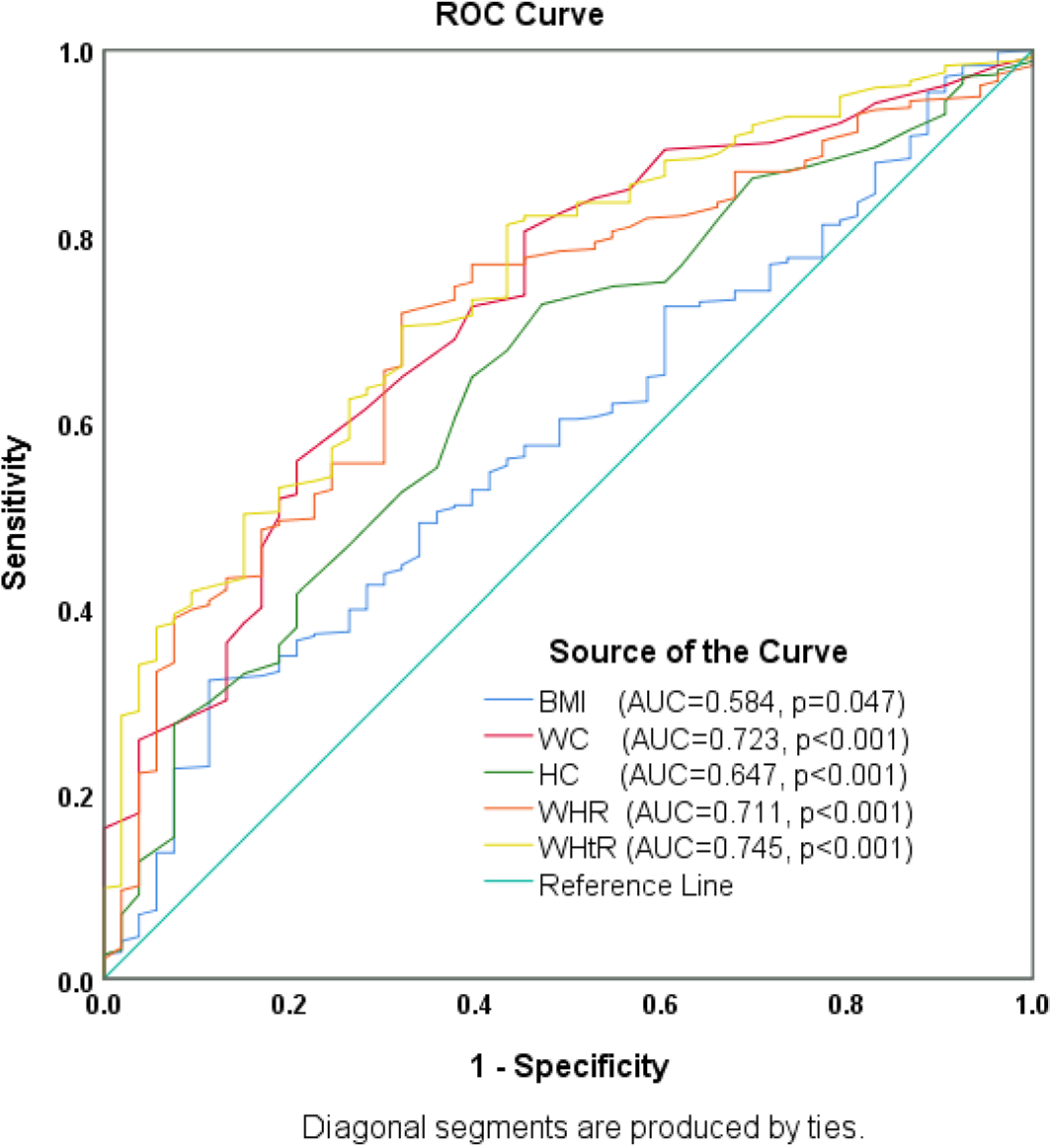
ROC curve analysis for comparison of BMI, WC, HC, WHR and WHtR in determining VF levels in diabetic patients.

The ROC curves showed that WHtR might be a better anthropometric index for identifying diabetic patients with high VF levels than other anthropometric indices. The areas under the ROC curves were as follows: WHtR (AUC = 0.75, 95% CI 0.68–0.81), BMI (AUC = 0.58, 95% Cl 0.51–0.66), WC (AUC = 0.72, 95% Cl 0.65–0.79), HC (AUC = 0.65, 95% Cl 0.57–0.72) and WHR (AUC = 0.71, 95% Cl 0.64–0.78) (see Fig. 1 and Table 3). The optimum cut-off values of > 25.7 kg/m^2^ for BMI (sensitivity, 60.2%; specificity, 49.1%), > 80.5 cm for WC (sensitivity, 72.4%; specificity, 39.6%), > 95.5 cm for HC (sensitivity, 67.6%; specificity, 43.4%), > 0.82 for WHR (sensitivity, 72.6%; specificity, 35.8%) and > 0.5 for WHtR (sensitivity, 67.6%; specificity, 32.1%) were determined by ROC curves to identify diabetic patients with high VF levels. The highest sensitivity (72.6%) for VF levels was shown by WHR, while the sensitivity of BMI (60.2%) was the lowest. Furthermore, the highest and lowest specificities were shown for BMI (49.1%) and WHtR (32.1%) (see Table 3).

**Table 3:** The AUC and the optimum cut-off points of anthropometric indices with VF levels in diabetic patients.

Test parameter	AUC	95% confidence intervals		*p* value	Optimum cut-off	Sensitivity	Specificity	Youden’s index
		Lower bound	Upper bound					
BMI	0.584	0.508	0.660	0.047	> 25.70	0.602	0.491	0.093
WC	0.723	0.653	0.793	< 0.001	> 80.50	0.724	0.396	0.120
HC	0.647	0.572	0.723	< 0.001	> 95.50	0.676	0.434	0.110
WHR	0.711	0.643	0.779	< 0.001	> 0.82	0.726	0.358	0.038
WHtR	0.745	0.680	0.809	< 0.001	> 0.50	0.676	0.321	0.003

## Discussion

Despite gender differences associated with diabetes incidence, some studies have proposed that BMI^2,4,10–12^, WC^2,3,11^, HC^1^ and WHR^2,10,20^ might be useful indices to predict adiposity, cardiovascular risk factors and metabolic syndrome. Studies have reported an association between high VF and the risk of diabetes complications, cardiometabolic diseases, and metabolic abnormalities in diabetic patients^2–4,10^; hence, assessing an individual’s body composition using appropriate indices is significant.

The findings of the present study showed that BMI, WC, HC, WHR and WHtR were statistically associated with VF levels determined by BIA among diabetic patients. The findings are useful when considering the burden of VF. Second, in the absence of BIA in low-resource settings for clinical practice and epidemiological studies, these anthropometric indices might be valuable for assessing VF levels. However, the regression analysis of the present study showed that WHtR [UOR = 21.49, *p* < 0.001] might be a better anthropometric index for identifying diabetic patients with high VF levels than BMI [UOR = 6.77, *p* = 0.008], WC [UOR = 6.37, *p* < 0.001], HC [UOR = 5.93, *p* = 0.002] and WHR [UOR = 13.17, *p* < 0.001] (see Table 2).

Likewise, the ROC curves of the anthropometric indices confirmed that WHtR showed the greatest predictive capacity of [AUC = 0.745, *p* ˂0.001] compared to BMI [AUC = 0.584, *p* = 0.047], WC [AUC = 0.723, *p* ˂0.001], HC [AUC = 0.647, *p* ˂0.001] and [AUC = 0.711, *p* ˂0.001] in identifying diabetic patients with high VF levels (see Table 3 and Fig. 1). The findings are innovative and added to the literature. For the first time, this study revealed the accuracy of using WHtR in identifying diabetic patients with high VF levels. It is, however, inconsistent with a study performed in Ghana by Eghan et al.^2^, who reported that both WC and HC had the largest ROC value [AUC = 0.79] in estimating VF among type II diabetic patients (T2DM) compared to BMI [AUC = 0.67], WHR [AUC = 0.53] and triceps skinfold thickness [AUC = 0.58]. The discrepancy could be the study setting and methodology.

There are inadequate studies performed for direct comparisons; however, many studies^3,4,10,20,21,26–28^, including two meta-analyses^17,18^ conducted globally in different study populations and ethnicities, have reported WHtR as the best anthropometric index. Those studies have focused on determining the accuracy of using anthropometric indices to predict adiposity, cardiometabolic risk factors and metabolic syndrome ^3,4,10,17,18,20,21,26–28^. Similar to the present study, although not a straightforward comparison, Moosaie et al.^4^ and Pasdar et al.^10^ reported that WHtR had the highest ROC values [AUC = 0.61 and 0.69] in predicting cardiovascular disease in T2DM patients and the healthy Iranian population, respectively. Dou et al.^29^ reported that WHtR had the maximum ROC values [AUC = 0.84 and 0.88] to predict cardiometabolic risk in Chinese children males and females. Tee et al.^27^ reported that WHtR had the greatest ROC values [AUC = 0.78 and 0.82] in predicting high blood pressure among Malaysian adolescent boys and girls. Shrestha et al.^28^ reported that WHtR had the highest ROC value [AUC = 0.60] as the best screening tool for hypertension among the Nepali population. Bacopoulou et al.^21^ reported that WHtR had the utmost ROC value [AUC = 0.97] in predicting abdominal obesity among Greek adolescents.

In contrast, again not a direct comparison with the present study, a study performed on the Iran population by Tutunchi et al.^22^ reported that both WHtR and WC had the highest ROC values [AUC = 0.97] in predicting overweight and obesity compared with WHR [AUC = 0.79]. In the Chinese population, Zhang et al.^3^ reported that both WC and WHtR had the greatest ROC values [AUC = 0.67 and AUC = 0.68, respectively] in predicting diabetes risk in Chinese males and females compared to BMI [AUC = 0.63] and WHR [AUC = 0.65]. Hernández-Vásquez et al.^14^ found that the conicity index had the maximum ROC value [AUC = 0.67] as the best predictor for diabetes in Peruvian men and women. However, the Ministry of Health in Peru has endorsed the use of both BMI and WC for the assessment of adiposity^14^.

The likely reasons are as follows: first, individuals with shorter heights have remarkably greater quantities of body fat compared to taller heights with the same BMI^4,14^. Second, individuals with similar WCs but different heights do not have the same quantities of body fat^3,4^. Third, being short in stature was associated with a higher accumulation of VF compared to being tall^3,14^. Additionally, studies have reported WC as a cardiometabolic risk factor compared to weight^4,14^. Finally, height alone predicts hypertension and diabetes, and the percentage of body fat associated with WC is an independent risk factor for cardiovascular disease^4^.

Although BMI also includes height measurement in the calculation, it is unable to differentiate between body fat and lean mass^2–4^ compared to WHtR, which is more reflective of body fat, particularly VF^2–4,10^. Studies have shown that BMI independently contributes to the prediction of VF^2,11,12^, which coincided with the present study. Therefore, BMI might be a possible index in identifying diabetic patients with high VF levels; however, the association of BMI and VF [UOR = 6.77, *p* = 0.008] and the precision [AUC = 0.584, *p* = 0.047] in identifying diabetic patients with high VF levels at cut-off > 25.7 kg/m^2^ were lower compared to WHtR (see Table 2, Table 3 and Fig. 1). The WHO has proposed that the BMI cut-offs at which substantial cardiometabolic risk is established vary depending on the country^10^. A prior study suggested that different BMI cut‐off points must be reviewed and reintroduced among ethnic populations for better sensitivity and specificity^10^.

Many studies have emphasized that WC might be a better index for cardiometabolic risk factors^3,4,10^. Other studies have reported an inverse association between height and cardiometabolic risk factors leading to morbidity and mortality^4,14^. The association of WC with VF [UOR = 6.37, *p* < 0.001] and the accuracy [AUC = 0.723, *p* ˂0.001] in identifying diabetic patients with high VF levels at cut-offs > 80.5 cm were lower than those of WHtR (see Tables 2, 3 and Fig. 1). Therefore, WC might be a possible index in identifying diabetic patients with high VF; however, it ignores the effect of height; hence, it might underestimate and overestimate the levels of VF in shorter and taller people. Furthermore, Hernández-Vásquez et al.^14^ reported that height is very important in some populations, especially Peruvian, who have shorter heights worldwide. In Peruvian adults, WHtR has been shown to have the strongest association with hypertension in both genders^14^.

Eghan et al.^2^ reported HC to be a potential predictor of VF estimates in diabetic patients, which is consistent with the present study. However, there were low to moderate associations between HC and VF levels compared to WHtR (see Table 2). The association of HC and VF [UOR = 5.93, *p* = 0.002] and the relative ability [AUC = 0.647, *p* ˂0.001] to correctly identify diabetic patients with high VF levels at cut-off > 95.5 cm was good; however, that of WHtR was better (see Table 2, Table 3 and Fig. 1). WHtR incorporates the height of the individual in the calculation, hence increasing the accuracy of the estimation of risks^14^. Additionally, Moosaie et al.^4^ reported WHtR as a more accurate tool for predicting hypertension in patients with T2DM.

Other studies have acknowledged WHR association with VF estimates^2^ and diabetes^3^; however, Eghan et al.^2^ have called for further study to appraise the efficacy of WHR. At a cut-off > 0.82 cm, WHR produced a relatively high sensitivity (72.6%) and a weak specificity of (35.8%), with [AUC = 0.711, *p* ˂0.001] (see Table 3 and Fig. 1). This implies that diabetic patients with higher WHR than normal are likely to have more accumulation of VF, which is risky for their health^2^. Based on the present study, WHR might be a possible index in identifying diabetic patients with high VF; however, the greater AUC for WHtR compared to WHR and its usefulness in diverse ethnicities^19,20^ recommend it as a better index for predicting adiposity.

WHtR showed higher efficacy than the other four anthropometric indices in identifying diabetic patients with high VF levels. First, it overpowered most of the limitations of BMI, WC, HC and WHR when adjusted (see Table 2). Second, WHtR has been recommended in several studies^3,4,10,20^ and provides a global cut-off value that is equally useful to both genders, ethnicities and different ages against other variables of abdominal adiposity^3,20,21,26^. Third, WHtR is shown to be a simple and reliable index to predict metabolic syndrome, cardiometabolic risk factors and adiposity at a cut-off > 0.5, which is the best index for identifying diabetic patients with high VF levels reported in the present study. These benefits have been brief in the following public health motto: “keep your waist circumference to less than half of your height”^10,20^. Finally, the advantages WHtR has over the four anthropometric indices are easiest to memorize for counselling patients, although it has no standard classification yet.

### Strengths and limitations

The strengths of this study exist in the study population, and it provides information for further study. Second, the measurements of the anthropometric variables were carried out by trained research assistants through dual assessments per a standard protocol to reduce recall and social desirability bias. Nonetheless, this study has some limitations. First, the diabetic patients were enrolled from one region out of sixteen regions in Ghana and limited to the selected hospitals; therefore, care should be taken when generalizing the findings. Second, information on diet, physical activity and lifestyle were not included in the analysis due to their scarcity; hence, adjusting for these covariates might affect the results of the regression analysis. Third, some of the biochemical and hemodynamic data were not available and recorded for all patients due to their appointment times. Furthermore, the five adiposity anthropometric indices were not analysed, presented and discussed according to gender using different cut-off values due to the insignificance of the analysed data according to gender. Finally, the use of BIA as the reference standard in this study was not classified according to gender^9^; therefore, care should be taken when deducing and generalizing the findings to the population.

## Conclusion

In the absence of BIA in low-resource settings for clinical practice and epidemiological studies, WHtR was shown to have overpowered BMI, HC, WC and WHR in identifying diabetic patients with high VF levels. Therefore, the Ghana Health Service could recommend WHtR as a better diagnostic index for assessing VF levels due to its high predictive capacity.

## Data Availability

The data used to support the findings of this study are available from the author upon request.

## Abbreviations

WHtR: Waist to height ratio
WHR: Waist to hip ratio
BMI: Body mass index
HC: Hip circumference
WC: Waist circumference
VF: Visceral fat
ROC: Receiver operating characteristics
UOR: Unadjusted odds ratio
AOR: Adjusted odds ratio
WHO: World Health Organization
CT: Computed tomography
MRI: Magnetic resonance imaging
BIA: Bioelectrical impedance analysis
Q: Quartile
OR: Odds ratio
FBS: Fasting blood sugar
SBP: Systolic blood pressure
DBP: Diastolic blood pressure
AUC: Area under the curve
T2DM: Type II diabetes mellitus

## References

[1] Agyemang-Yeboah F, Eghan BAJ, Annani-Akollor ME, Togbe E, Donkor S, Oppong AB (2019). Evaluation of metabolic syndrome and its associated risk factors in type 2 diabetes: A descriptive cross-sectional study at the Komfo Anokye teaching hospital, Kumasi, Ghana. Biomed. Res. Int..

[2] Eghan BA, Agyemang-Yeboah F, Togbe E, Annani-Akollor ME, Donkor S, Afranie BO (2019). Waist circumference and hip circumference as potential predictors of visceral fat estimate among type 2 diabetic patients at the Komfo Anokye teaching hospital (KATH) Kumasi-Ghana. Alex. J. Med..

[3] Zhang F-L, Ren J-X, Zhang P, Jin H, Qu Y, Yu Y (2021). Strong Association of waist circumference (WC), body mass index (BMI), waist-to-height ratio (WHtR), and waist-to-hip ratio (WHR) with diabetes: A population-based cross-sectional study in Jilin province, China. J. Diabetes Res..

[4] Moosaie F, Abhari SMF, Deravi N, Behnagh AK, Esteghamati S, Firouzabadi FD (2021). Waist-to-height ratio is a more accurate tool for predicting hypertension than waist-to-hip circumference and BMI in patients with type 2 diabetes: A prospective study. Front. Public. Heal..

[5] Kuwabara M, Kuwabara R, Niwa K, Hisatome I, Smits G, Roncal-Jimenez CA (2018). Different risk for hypertension, diabetes, dyslipidemia, and hyperuricemia according to level of body mass index in Japanese and American subjects. Nutrients.

[6] Perona JS, Schmidt Rio-Valle J, Ramírez-Vélez R, Correa-Rodríguez M, Fernández-Aparicio Á, González-Jiménez E (2019). Waist circumference and abdominal volume index are the strongest anthropometric discriminators of metabolic syndrome in Spanish adolescents. Eur. J. Clin. Invest..

[7] Omura-Ohata Y, Son C, Makino H, Koezuka R, Tochiya M, Tamanaha T (2019). Efficacy of visceral fat estimation by dual bioelectrical impedance analysis in detecting cardiovascular risk factors in patients with type 2 diabetes. Cardiovasc. Diabetol..

[8] Muhanna, R. G, Aljuraiban, G. S., Almadani, N. K., Alquraishi, M., El-Sharkawy, M. S., Abulmeaty, M. M. A. Value of adding bioelectrical impedance analysis to anthropometric indices in the diagnosis of metabolic syndrome in 10–16 years old schoolgirls. In: *Healthcare. MDPI*; p. 1–10 (2022). 10.3390/healthcare10030419 PMC8950802 35326897

[9] Omron Healthcare Co. Ltd. BF511 OJIM, all for healthcare. Body Composite Monitor. 3 (2015).

[10] Pasdar Y, Moradi S, Moludi J, Saiedi S, Moradinazar M, Hamzeh B (2020). Waist-to-height ratio is a better discriminator of cardiovascular disease than other anthropometric indicators in Kurdish adults. Sci. Rep..

[11] Li N, Yang T, Yu W-Q, Liu H (2019). Is waist-to-height ratio superior to body mass index and waist circumference in predicting the incidence of hypertension?. Ann. Nutr. Metab..

[12] Camhi SM, Bray GA, Bouchard C, Greenway FL, Johnson WD, Newton RL (2011). The relationship of waist circumference and BMI to visceral, subcutaneous, and total body fat: Sex and race differences. Obesity.

[13] Lu Y, Liu S, Qiao Y, Li G, Wu Y, Ke C (2021). Waist-to-height ratio, waist circumference, body mass index, waist divided by height0.5 and the risk of cardiometabolic multimorbidity: A national longitudinal cohort study. Nutr. Metab. Cardiovasc. Dis..

[14] Hernández-Vásquez A, Azañedo D, Vargas-Fernández R, Aparco JP, Chaparro RM, Santero M (2021). Cut-off points of anthropometric markers associated with hypertension and diabetes in Peru: Demographic and Health Survey 2018. Public Health Nutr..

[15] World Health Organization. Nutrition https://www.euro.who.int/en/health-topics/disease-prevention/nutrition/a-healthy-lifestyle/body-mass-index-bmi. World Health Organization 2021. 2021;:Accessed February 8, 2022.

[16] World Health Organization WHO. Waist circumference and waist to hip ratio: Report of a WHO expert consultation geneva 2011 WHO. https: www.who.int/ nutrition publications/obesity/WHO_report_waist circumference_and_waisthipratio/en/ (accessed March 2021). (2021).

[17] Deng, G., Yin, L., Liu, W., Liu, X., Xiang, Q., Qian, Z., *et al.* Associations of anthropometric adiposity indexes with hypertension risk: A systematic review and meta-analysis including PURE-China. *Medicine (Baltimore)*. **97**, (2018). 10.1097/MD.0000000000013262 PMC6283208 30508913

[18] Jiang Y, Dou Y, Chen H, Zhang Y, Chen X, Wang Y (2021). Performance of waist-to-height ratio as a screening tool for identifying cardiometabolic risk in children: A meta-analysis. Diabetol. Metab Syndr..

[19] Corrêa MM, Thumé E, De Oliveira ERA, Tomasi E (2016). Performance of the waist-to-height ratio in identifying obesity and predicting non-communicable diseases in the elderly population: A systematic literature review. Arch Gerontol. Geriatr..

[20] Ashwell M, Gibson S (2016). Waist-to-height ratio as an indicator of ‘early health risk’: Simpler and more predictive than using a ‘matrix’based on BMI and waist circumference. BMJ Open.

[21] Bacopoulou F, Efthymiou V, Landis G, Rentoumis A, Chrousos GP (2015). Waist circumference, waist-to-hip ratio and waist-to-height ratio reference percentiles for abdominal obesity among Greek adolescents. BMC Pediatr..

[22] Tutunchi H, Ebrahimi-Mameghani M, Ostadrahimi A, Asghari-Jafarabadi M (2020). What are the optimal cut-off points of anthropometric indices for prediction of overweight and obesity? Predictive validity of waist circumference, waist-to-hip and waist-to-height ratios. Heal. Promot. Perspect..

[23] Tuglo LS, Nyande FK, Agordoh PD, Nartey EB, Pan Z, Logosu L (2022). Knowledge and practice of diabetic foot care and the prevalence of diabetic foot ulcers among diabetic patients of selected hospitals in the Volta region. Ghana. Int Wound J..

[24] Tuglo LS (2022). Prevalence and determinants of lower extremity amputations among type I and type II diabetic patients: A multicenter-based study. Int. Wound J..

[25] Tuglo LS, Agbadja C, Bruku CS, Kumordzi V, Tuglo JD, Asaaba LA (2022). The association between pregnancy-related factors and health status before and after childbirth with satisfaction with skilled delivery in multiple dimensions among postpartum mothers in the Akatsi South district, Ghana. Front. Public Heal..

[26] Cho S, Shin A, Choi J-Y, Park SM, Kang D, Lee J-K (2021). Optimal cutoff values for anthropometric indices of obesity as discriminators of metabolic abnormalities in Korea: Results from a health examinees study. BMC Public Health.

[27] Tee JYH, Gan WY, Lim PY (2020). Comparisons of body mass index, waist circumference, waist-to-height ratio and a body shape index (ABSI) in predicting high blood pressure among Malaysian adolescents: A cross-sectional study. BMJ Open.

[28] Shrestha R, Upadhyay SK, Khatri B, Bhattarai JR, Kayastha M, Upadhyay MP (2021). BMI, waist to height ratio and waist circumference as a screening tool for hypertension in hospital outpatients: A cross-sectional, non-inferiority study. BMJ Open.

[29] Dou Y, Jiang Y, Yan Y, Chen H, Zhang Y, Chen X (2020). Waist-to-height ratio as a screening tool for cardiometabolic risk in children and adolescents: A nationwide cross-sectional study in China. BMJ Open.

